# Exploring the Thermal and Mechanical Properties of Thermoset-Based Composites Reinforced with New Continuous and Chopped Phosphate Glass Fibers

**DOI:** 10.3390/polym17121627

**Published:** 2025-06-11

**Authors:** Iliass Daki, Nezha Saloumi, Mohamed Yousfi, Caroline Parajua-sejil, Vivien Truchot, Jean-François Gérard, Omar Cherkaoui, Hassan Hannache, Mehdi El Bouchti, Mina Oumam

**Affiliations:** 1 REMTEX Laboratory, Higher School of Textile and Clothing Industries (ESITH), Casablanca 20200, Morocco; 2 Laboratory of Engineering and Materials LIMAT, Faculty of Science Ben M’Sik, Hassan II University, Casablanca 20670, Morocco; 3 Université de Lyon, CNRS, UMR 5223, Ingénierie des Matériaux Polymères, Université Claude Bernard Lyon 1, INSA Lyon, Université Jean Monnet, F-69621 Villeurbanne, France; 4 Materials Science and Nanoengineering Department, Mohamed VI Polytechnic University, Benguerir 43150, Morocco

**Keywords:** phosphate glass fibers, thermoset matrices, contact molding, physical, thermal and mechanical properties

## Abstract

Currently, the main drivers for the production of phosphate glass fiber-reinforced composites are the growing demand for lightweight materials, reduced energy consumption, improved durability, and minimized environmental impact. This study aims to develop thermoset-based composites using chopped and continuous phosphate glass fibers (PGFs) combined with polyester and epoxy matrices, processed via contact molding. Physical, mechanical, thermal, and morphological characterizations were conducted. The addition of PGFs led to a steady increase in density and fiber volume fraction. For polyester composites with short PGFs, density rose from 1.60 g/cm^3^ (0 wt%) to 1.77 g/cm^3^ (22.8 wt%), with a corresponding volume fraction increase from 0% to 14.4%. Similarly, epoxy composites showed density values from 1.70 g/cm^3^ to 1.87 g/cm^3^ and volume fractions up to 15.2%. Thermogravimetric analysis (TGA) showed that as the fiber content increased, the thermal degradation of the resin was delayed, as evidenced by a rise in onset degradation temperature and greater residual mass—indicating improved thermal stability of the composites. Tensile strength increased from 20.8 MPa to 71.3 MPa (polyester) and from 26.8 MPa to 75.9 MPa (epoxy) with chopped fibers, reaching 145.7 MPa and 187.9 MPa, respectively, with continuous fibers. Flexural strength reached 167.9 MPa (polyester) and 218.0 MPa (epoxy) in continuous-fiber configurations. Young’s modulus values closely matched Hirsch model predictions. These findings confirm the potential of PGF-reinforced thermoset composites for high-performance and sustainable material applications.

## 1. Introduction

The advance of organic matrix composite materials has taken a leading role in materials engineering, offering innovative prospects for the development of lightweight, high-strength structures [1]. Composite materials are emerging as the materials of the century, thanks to their excellent insulating properties, their high resistance to fatigue and corrosion, and their increasingly affordable costs, based on their high consumption and mastery of processing [2]. They are used in fields as varied as aerospace, aeronautics, car manufacturing, transport, shipbuilding, biomedical, petrochemicals, and civil engineering [3].

The market for composite materials is currently experiencing growing demand, stimulated by the ongoing search for innovative solutions in various industrial sectors [4,5]. The integration of glass fibers in thermoset matrix composites meets the essential requirements of today’s market, particularly in the aerospace sector [6]. The need to reduce component weight while maintaining optimum structural strength is crucial in this field. Glass fiber composites are particularly attractive, offering exceptional lightness while maintaining high mechanical strength. This can help improve aircraft fuel efficiency and reduce environment impact, making them a suitable choice [7]. In the automotive industry, the increasing desire for lighter vehicles to decrease emissions has propelled composite materials to the forefront of innovation [8].

Composites based on phosphate glass fibers with a thermosetting matrix are therefore part of this trend, offering an environmentally friendly option without compromising on quality [9]. Thanks to their unique properties, phosphate gives glass fibers exceptional strength and remarkable lightness, making them an ideal choice for applications requiring both strength and lightness. Phosphate glass fibers demonstrate chemical stability, ensuring resistance to chemical reactions and bolstering durability in aggressive chemical settings [10,11]. Additionally, they display commendable heat resistance, rendering them suitable for applications in elevated-temperature environments [12]. While possessing high tensile strength, glass fibers exhibit relative weaknesses in compression and shear. When integrated with a polymer resin, these materials create structural composite components that excel in withstanding compression, tension, and bending forces [13]. It is important to note that the properties mentioned can vary based on the specific formulation of the phosphate glass fibers [13]. These characteristics are further enhanced when combined with a thermosetting matrix. The inherent thermal stability of the thermosetting matrix makes it an ideal companion for phosphate glass fibers. During the curing process, the thermoset matrix forms a stable three-dimensional structure that considerably improves the overall strength of the composite material. This synergistic marriage of phosphate glass fibers and thermoset matrix results in polymer composites with superior mechanical properties that withstand extreme thermal conditions [14]. In this context, the incorporation of fibers into composite materials plays a key role in enhancing mechanical performance, especially in tensile, flexural, and compressive strength. Yang et al. demonstrated that glass fibers act as crack arresters and stress transmitters, significantly improving the structural behavior of phosphate-activated geopolymer composites [15].

Furthermore, recent advancements show that combining nanotechnology with fiber-reinforced polymers can further enhance their mechanical, thermal, and multifunctional performance. As noted by Rashid, nanomaterials like carbon nanotubes and nanoclays improve stress transfer and interfacial bonding, thus enabling the use of such materials in demanding applications like aerospace and automotive industries [16].

From a manufacturing perspective, composite integrity is also influenced by post-processing techniques such as drilling. Bekir Yalçın revealed that optimizing drilling parameters (spindle speed, feed rate, tool diameter) reduces delamination and improves the structural quality of high-performance glass/epoxy composites, an insight valuable for aerospace applications [17].

Another avenue for performance enhancement is surface modification of glass fibers. Yawen Wu’s review shows that chemically modified glass fibers exhibit improved wettability, mechanical strength, and multifunctionality. However, challenges remain in optimizing modification techniques, promoting eco-friendly processing, and managing end-of-life strategies for sustainable use [18].

Finally, the choice of matrix has a decisive impact on the final composite performance. Polyester and epoxy resins have emerged as more versatile and practical alternatives to phenolic resins. Despite their superior thermal stability, phenolic resins suffer from brittleness and difficult processing. In contrast, epoxy resins offer superior adhesion, chemical resistance, and strength, while polyester resins provide cost-efficiency and faster curing. These advantages make polyester and epoxy ideal candidates for structural composites in automotive, aerospace, and marine sectors [19].

The present study aims to evaluate the potential of phosphate glass fibers (PGFs) as a reinforcing phase to be used in thermoset-based composites, using a high-performance glass formulation (52P_2_O_5_–24CaO–13MgO–5Fe_2_O_3_–1TiO_2_–5Al_2_O_3_). The latter was selected based on a comparative study among various phosphate glass formulations and specifically designed to overcome the conventional limitations of phosphate glasses for ultimate mechanical applications. Despite the frequent classification of phosphate glasses as weak reinforcements due to limited durability and strength, the present optimized composition (52P_2_O_5_–24CaO–13MgO–5Fe_2_O_3_–1TiO_2_–5Al_2_O_3_) offers improved chemical durability, mechanical integrity, and thermal stability. This composition was designed to balance bioactivity, thermal stability, and mechanical integrity. Specifically, it avoids alkali content, which is typically responsible for poor chemical durability, and includes Fe_2_O_3_ and TiO_2_ to enhance the network connectivity and degradation resistance compared to traditional phosphate glasses.

The study aims to evaluate the improvement of the thermal stability and structural integrity of phosphate glass fiber-reinforced composites, by investigating the influence of fiber morphology (chopped vs. continuous) and resin type (polyester vs. epoxy). The work focuses on the fabrication and characterization of cut and continuous PGF-reinforced composites via contact molding, with the goal of demonstrating that, through compositional design and appropriate processing, PGFs can rival conventional reinforcement fibers such as E-glass, thereby contributing to the development of sustainable and efficient composite materials.

## 2. Materials and Methods

### 2.1. Process for the Preparation of Composites Reinforced with Phosphate Glass Fibers

#### 2.1.1. Preparation of Phosphate Glass Reinforcement

Phosphate glasses of the 52P_2_O_5_-24CaO-13MgO-5Fe_2_O_3_-1TiO_2_-5Al_2_O_3_ system were produced using the direct raw material fusion method. The precursors (in powder form) included calcium phosphate dibasic dihydrate (CaH_3_PO_4_), magnesium oxide (MgO), titanium oxide (TiO), iron (III) chloride hexahydrate (FeCl_3_), and aluminum chloride (Al_2_Cl_3_) in appropriate proportions. These raw materials were weighed and premixed in a crucible, then placed in an electric furnace, heated to 300 °C for 1 h to remove H_2_O and CO_2_, then the temperature was slowly increased to 1200 °C. The molten glass was then left to cool to room temperature. Phosphate glass fiber was produced by the melt-drawing method. Treated phosphate glass was drawn at a spinning speed of 500 m/min and at a spinning temperature up to 680 °C. Using an optical microscope, the diameter of the resulting fiber was presented at an average of 11 ± 0.5 μm. The characteristics of phosphate glass fibers are detailed in Table 1.

It is worth noting that the tensile strength value reported in Table 1 (2668 MPa) exceeds the typical range generally reported in the literature for conventional phosphate glass fibers (approximately 500–1500 MPa) [20]. This result stems from a targeted design approach aimed at enhancing the mechanical performance of phosphate glass fibers. Specifically, the partial substitution of K_2_O by a combination of Al_2_O_3_, Fe_2_O_3_, and TiO_2_ in the glass composition led to the formation of P–O–M (M = Al, Fe, Ti) covalent bonds, known to strengthen the glass network and increase crosslinking. This strategy resulted in a significant increase in glass density (up to 2.80 g/cm^3^), glass transition temperature (Tg), and mechanical strength [20]. The optimized formulation (52P_2_O_5_–24CaO–13MgO–5Al_2_O_3_–5Fe_2_O_3_–1TiO_2_) allowed for achieving a tensile strength of 2668 MPa and an elastic modulus of 140 GPa. These values were supported by statistical correlation analysis between glass composition and measured properties.

#### 2.1.2. Matrix Preparation

Thermosetting resins are widely used in composites because of their chemical resistance, ease of handling, low cost, and rapid curing without gas emissions. During the curing process, the polymer is referred to as a resin system, and after it has dried, it is referred to as a matrix, characterized by low viscosity. In this respect, we have chosen polyester or epoxy resin as the matrix. Thermosetting resins are liquid materials; to convert to a solid state, a new chemical bond must be created. However, this can only begin if the temperature is 80 °C or higher. For the resin to cure at a temperature of 25 °C, it is necessary to add a gas pedal and a catalyst, respectively.

- Polyester matrix

The unsaturated polyester resin curing process requires the addition of a catalyst (methyl ethyl ketone peroxide) and an accelerator (cobalt) to speed up the resin curing process. These components were purchased from Detail Chimie in Casablanca, Morocco. In the present study, a resin formulation comprising 2% methyl ethyl ketone peroxide and 0.24% cobalt gas pedal was used.

b. Epoxy matrix

The epoxy resin is easy to handle and polymerization can take place at room temperature, depending on the hardener chosen and the weight ratio. Epoxy and hardener were also obtained from detail Chimie (Casablanca, Morocco). The epoxy matrix is prepared by incorporating amine-based hardeners, acting as curing agents, with a weight distribution of 66% epoxy and 33% hardener.

#### 2.1.3. Elaboration of Composite Specimens

Thermoset composites reinforced with phosphate glass fibers were produced using the contact molding technique at a temperature of 25 °C. To achieve random orientation, phosphate glass fibers were cut into 2 ± 1 mm segments and randomly distributed to create cut geometries. The resins were prepared in an open container by adding the specific proportions of catalyst and hardener previously defined. The mixture was then mechanically stirred at a speed of 900 rpm for 5 min, with the gradual addition of fibers during stirring. Finally, the resulting mixture was poured into a steel mold treated with a release wax, facilitating demolding of the composites.

**Figure 1:**
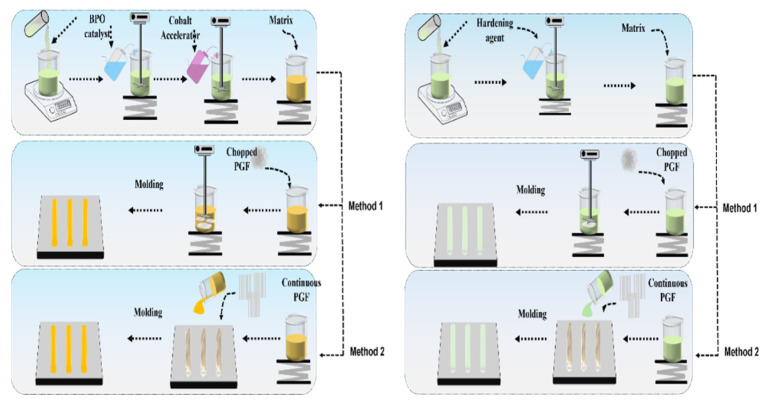
Composite production process of thermoset-based composites, whether short-fiber or continuous.

Continuous phosphate glass fiber-based composites were made by selecting a small part of fibers from the main bundle, then aligning them in a parallel direction. The same previous resin preparation was used for these composites. The fibers were carefully arranged in the steel mold, then the resin was poured in slowly to ensure adequate infiltration, with the help of a lamination roller to eliminate air bubbles. After 24 h, the resin cured, forming the final composite with dimensions conforming to ISO 527 and ISO 14125 standards [21,22]. All composites were then placed in an oven at 80 °C for 24 h to enhance curing. Figure 1 illustrates the manufacturing process for thermoset composites, whether short-fiber or continuous.

### 2.2. Characterization of Composites Reinforced with Phosphate Glass Fibers

In this study, we investigated the physical properties of the developed composites by determining key parameters such as mass fraction, volume fraction, fiber volume, and composite density. The mass fraction (noted as *V_m_*) expresses the weight proportion of the reinforcement in the composite and is calculated using the equation

(1) $$V_{m}=\frac{m_{r}}{m_{c}}\;\%$$

where *m_r_* is the mass of the reinforcement (fiber) and *m_c_* is the total mass of the composite. This parameter is useful for comparing different formulations and ensuring process consistency. To evaluate the fiber content independently of its mass, we calculated the fiber volume (*V_r_*) based on the equation

(2) $$V_{r}=\frac{m_{r}}{\rho }$$

where *ρ* is the density of the reinforcement (2.8 g/cm^3^). This volume is essential to determine the volume fraction (*V_f_*) of the fibers in the composite using

(3) $$V_{f}=\frac{V_{r}}{V_{c}}\;\%$$

where *Vc* is the total volume of the composite.

The composite volume was obtained geometrically from its dimensions, using the relation

(4) $$V_{c}=L*l*e$$

where *L*, *l*, and *e* represent the length, width, and thickness of the composite plate, respectively.

Finally, the density of the composite (*ρ_c_*) was calculated as

(5) $$\rho =\frac{m_{c}}{V_{c}}\;$$

This final value allows the assessment of fabrication quality by detecting porosity or poor fiber wetting. Altogether, these calculations provide a clear interpretation of the mass and volume contributions of the fibers, helping to evaluate the structural efficiency and consistency of the composite manufacturing process.

Thermogravimetric analysis (TGA) was conducted using a Mettler Toledo instrument, Greifensee, Switzerland, to examine the thermal stability of the samples. All analyses were performed on samples weighing 5.4 mg, subjected to a temperature ramp of 10 °C/min up to 650 °C, under a nitrogen atmosphere. Furthermore, mechanical properties were evaluated through tensile and bending tests. The latter, including the three-point bending test and the tensile test, served to measure the material’s resistance to fracture. Flexural and tensile analysis of the developed composites was carried out using the Zwick Roell machine (Kronberg im Taunus, Germany), adhering to ISO 527 (tensile) and ISO 14125 (flexural) standards, with a load application speed of 2 mm/min. The morphology of the composites was examined using a JEOL JCM 6000Plus scanning electron microscope (Tokyo, Japan) to assess their quality. Additionally, the quality of composite consolidation was investigated by observing polished and unpolished cross-sections of fractured samples after mechanical testing. To enhance the quality of SEM micrographs, all samples underwent metallization by coating them with a thick layer of carbon before analysis. Results for physical properties (such as density, mass fraction, and volume fraction) and mechanical properties were obtained from five samples tested under the same conditions, in order to guarantee the repeatability and reliability of experimental measurements.

## 3. Results and Discussion

### 3.1. Physical Properties

The development of a polyester or epoxy matrix composite reinforced with phosphate glass fibers and the analysis of its density depend on various factors, including the reinforcement/resin mix, the geometry of the reinforcements, and the presence of voids. These factors reveal significant trends in our study. The density of the matrix, composed of polyester and epoxy, was evaluated independently, highlighting the potential dominance of epoxy, often associated with a higher density than polyester. Examination of the data in Table 2 and Table 3 shows that the progressive integration of glass fibers, chopped (denoted S-PGF) or continuous (denoted L-PGF), led to a general increase in density, in line with the expected impact of reinforcements, with an average fiber density of 2.8 g/cm^3^. Phosphate glass chopped fibers, in particular, significantly increased density, from 1.6 g/cm^3^ to 1.77 g/cm^3^ for polyester and from 1.7 g/cm^3^ to 1.87 g/cm^3^ for epoxy. For continuous geometries, the uniform distribution of these fibers also increased composite density from 1.64 g/cm^3^ to 1.71 g/cm^3^ for polyester and from 1.74 g/cm^3^ to 1.8 g/cm^3^ for epoxy, a trend consistent with the expected effects of reinforcements. However, this analysis becomes even more complex when considering the two glass fiber geometries [23,24]. The distribution of continuous fibers in the matrix can also reduce porosity, thus increasing density without compromising mechanical properties. Chopped glass fibers, due to their greater specific surface area, may have a different impact on density than continuous fibers, which contribute more to the materials rigidity [25].

### 3.2. Thermal Properties

**Figure 2:**
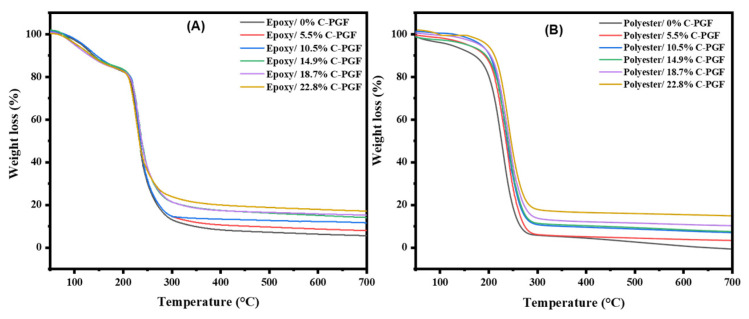
TGA data for composites based on chopped phosphate glass fibers and thermosetting epoxy (**A**) and polyester (**B**) resins.

**Figure 3:**
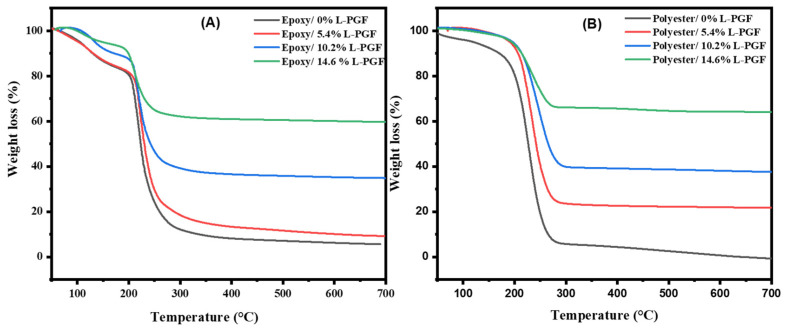
TGA data for composites based on continuous phosphate glass fibers and “epoxy (**A**) and polyester (**B**)” thermosetting resins.

Thermogravimetric analysis was used to assess the thermal stability of composites made from phosphate glass fiber-reinforced polyester or epoxy resins. The TGA curves for all samples are shown in Figure 2 and Figure 3, while the degradation ranges are summarized in Table 4 and Table 5.

For the polyester matrix without glass fibers (0%), the thermal behavior of pure polyester is observed, with a significant weight loss around 250 °C. The incorporation of chopped glass fibers into the polyester resin significantly improves thermal stability. As the fiber content increases (from 0 to 22.8%), the thermal degradation of the polyester matrix is delayed, as evidenced by an increase in the onset degradation temperature and a higher residual mass (see Table 4 and Table 5), indicating improved thermal stability of the composite. When continuous glass fibers were incorporated into the polyester resin, further improvement in thermal stability was observed. This observation can be attributed to the fact that continuous glass fibers provide greater mechanical reinforcement than chopped fibers, thereby strengthening the composite structure and giving it greater thermal resistance. Additionally, continuous glass fibers act as a more effective physical barrier against thermal shocks, thus delaying the thermal degradation of the resin. Furthermore, the larger specific surface area offered by continuous glass fibers promotes better adhesion between the fibers and the resin, which enhances stress transfer and the overall thermal resistance of the composite. Similar to the epoxy-based composites, the addition of chopped phosphate fibers also improved thermal stability (see Table 4 and Table 5). The phosphate fibers act as reinforcing agents, contributing to a more rigid structure and offering better resistance to thermal shocks. Moreover, the interaction between the fibers and the matrix promotes better adhesion and improves stress transfer.

It is also important to note that the maximum degradation temperature (Tmax) for polyester composites with 22.8% phosphate fibers reaches up to 329 °C, whereas the Tmax for the epoxy matrix under the same conditions is significantly lower (235 °C). This difference can be attributed to the inherent differences between the two resins: the polyester resin, having a lower viscosity, may allow better wetting and impregnation of the phosphate fibers, leading to improved fiber–matrix interaction. Additionally, specific interactions between the resin’s functional groups and the phosphate network could enhance thermal stability in the polyester system more effectively than in epoxy.

On the other hand, it is observed that modifying the fiber size increases the degradation temperature of the epoxy resin, which can be explained based on the same assumptions proposed when interpreting the results for polyester resin. Comparing the results in the tables for epoxy and polyester composites reinforced with chopped and continuous phosphate glass fibers, it appears that the addition of phosphate glass fibers and the modification of their geometry generally improve the thermal stability of the composites, particularly by increasing the amount of residue after degradation. Epoxy composites, in particular, show a clear improvement in thermal stability, with significantly higher residues, especially in the samples containing continuous fibers.

However, despite the improved residual mass, the maximum degradation temperature (Tmax) for epoxy composites remains below 250 °C for all compositions studied, indicating that their thermal resistance plateau is lower than that of polyester-based composites with higher fiber contents. Therefore, the conclusion regarding the thermal stability must consider this upper temperature limitation. This indicates that, although phosphate glass fibers improve overall thermal resistance, their impact on the maximum degradation temperature must be carefully optimized for each type of matrix [26].

In comparison with previous works in the literature, it is observed that the thermal stability of glass fiber-reinforced thermoset composites is influenced by various factors, including the proportion of glass fibers. Budai et al. examined the thermal behavior of a glass fiber-reinforced unsaturated polyester composite, featuring different configurations in terms of the number of glass mat layers (4, 6, and 11 layers), through TGA analyses. They found that increasing the glass fiber content retarded thermo-oxidative decomposition [27]. On the other hand, Hameed et al. studied the thermal performance of modified epoxy composites reinforced with chopped strands of glass fibers, with various fiber proportions ranging from 10% to 60%. Their thermogravimetric analysis (TGA) indicated that composites with a fiber content of 60% exhibited enhanced thermal stability, characterized by an increase in degradation temperature from 357 °C to 390 °C [28].

### 3.3. Mechanical Properties

#### 3.3.1. Tensile Testing

**Figure 4:**
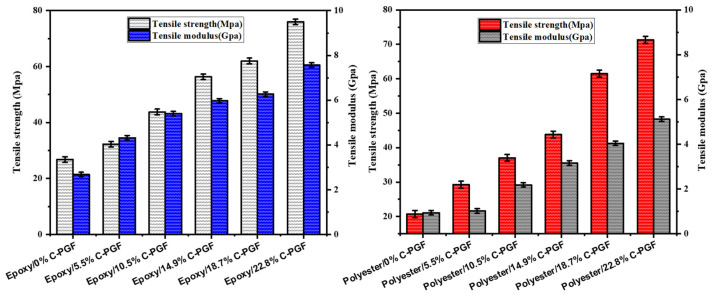
Effect of chopped phosphate glass fiber content (in weight percent) on tensile strength and modulus of epoxy and polyester resin.

**Figure 5:**
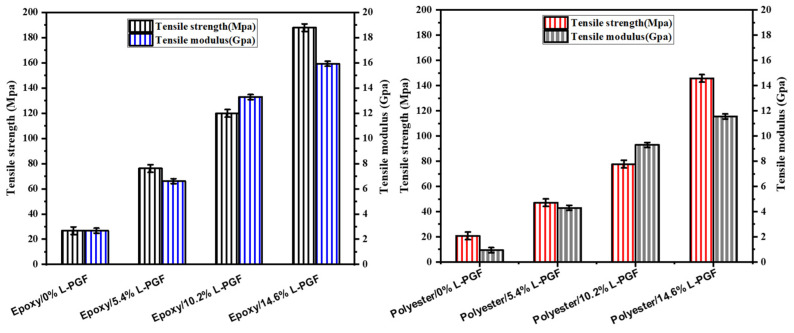
Effect of continuous phosphate glass fiber content (in weight percent) on tensile strength and modulus of epoxy and polyester resin.

Figure 4 shows the influence of fiber quantity on the tensile strength of composites made from a thermosetting resin reinforced with phosphate glass fibers in a cut geometry. Similarly, Figure 5 shows the influence of the amount of fiber in a continuous geometry on the tensile strength of the corresponding composites. The data reveal a significant increase in tensile strength with increasing mass fraction of phosphate glass fiber composites, for both polyester and epoxy in cut geometry. For polyester, tensile strength increases from 20.8 MPa to 71.3 MPa, while for epoxy, it rises from 26.8 MPa to 75.9 MPa. This improvement in mechanical properties suggests that both resins benefit from the addition of phosphate glass fibers, although polyester shows a proportionally greater increase than epoxy. In a continuous geometry, both resins also show significant increases in tensile strength with increasing composite mass fraction. For polyester, tensile strength increases from 20.8 MPa to 145.7 MPa, while for epoxy, it rises from 26.8 MPa to 187.9 MPa. These results underline the significant impact of the addition of phosphate glass fibers on tensile strength, with generally higher absolute values for epoxy, indicating a better overall performance of this resin in this context. The differences between polyester and epoxy resins also lie in their mechanical properties, with epoxy-based composites generally being stiffer and more tensile-resistant, with better fatigue resistance and fiber adhesion than polyester-based composites. In addition, epoxy offers greater chemical resistance and durability, although it is generally more expensive and requires more rigorous curing conditions than polyester, which can be cured at room temperature with the aid of catalysts.

#### 3.3.2. Flexural Testing

**Figure 6:**
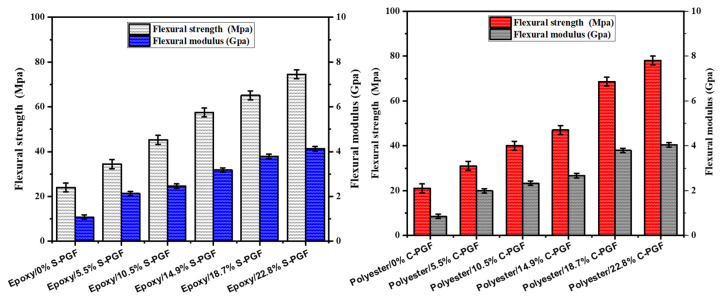
Effect of chopped phosphate glass fiber content (in weight percent) on flexural strength and modulus of epoxy and polyester resin.

**Figure 7:**
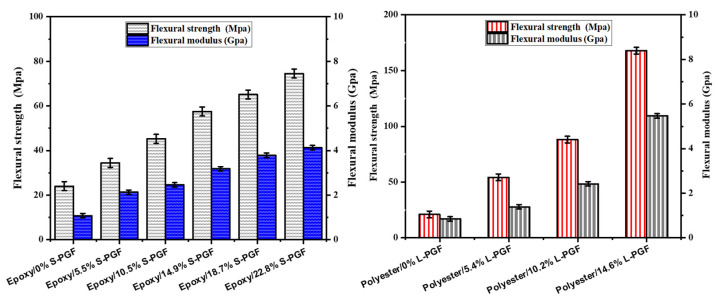
Effect of continuous phosphate glass fiber content (in weight percent) on flexural strength and modulus of epoxy and polyester resin.

Figure 6 shows how the amount of fiber affects the flexural strength of composites, with a chopped geometry, made from phosphate glass fiber-reinforced thermosetting resin. Similarly, Figure 7 examines how this amount of fiber affects the flexural strength of the same composites, but with a continuous geometry. The data provided show a significant increase in flexural strength as the mass fraction of polyester and epoxy composites increases, in both chopped and continuous geometries. In the cut geometry, for polyester, flexural strength rises from 21.0 MPa to 78.0 MPa as the mass fraction increases from 0% to 22.8%. For epoxy in the same geometry, flexural strength increases from 24.0 MPa to 74.6 MPa over the same mass fraction range. However, in a continuous geometry, both resins show a similar trend of increasing flexural strength with increasing mass fraction. For polyester, flexural strength increases from 21.0 MPa to 167.9 MPa when the mass fraction increases from 0% to 14.6%, while for epoxy, it increases from 24.0 MPa to 218.0 MPa in the same mass fraction range. These results indicate that the addition of phosphate glass fibers improves the flexural strength of composites for both resins and in both geometries, although epoxy shows a more significant increase in flexural strength, particularly in a continuous geometry, suggesting a better suitability of epoxy for applications requiring high flexural strength.

From our study and Table 6, we can say that the mechanical properties of composites are influenced by various factors, among which fiber content and geometry play a prominent role. Firstly, fiber content, generally expressed as a percentage of volume (V_f_), directly affects the composite’s strength and stiffness. A higher proportion of fibers generally translates into increased mechanical strength, as the fibers provide a direct load path and prevent crack propagation. However, excessive fiber content can also lead to a reduction in the ductility and toughness of the composite material due to poor adhesion between matrix and fibers, as well as the formation of defects such as voids. Fiber geometry, including size, shape and arrangement, is crucial [29]. Optimized geometry promotes better dispersion of the fibers in the matrix, resulting in a more homogeneous distribution of fillers and improved overall mechanical properties of the composite. Smaller fiber sizes or better fiber orientation can also enhance strength and stiffness, while excessive agglomeration or poor fiber distribution can lead to weak zones and reduced mechanical performance [30].

### 3.4. Morphological Properties

**Figure 8:**
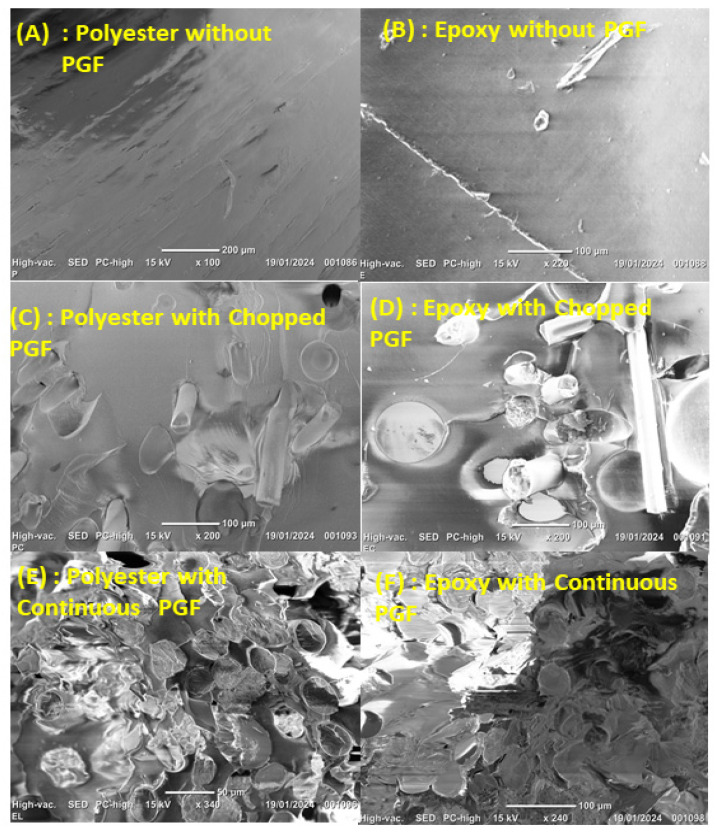
SEM images of tensile fracture surfaces of composite materials.

The interfacial bond strength between the thermosetting matrices and the phosphate glass fibers (PGFs), along with the morphology of the fracture surfaces following mechanical testing, was investigated using scanning electron microscopy (SEM), as illustrated in Figure 8A–F.

Figure 8 SEM micrographs of fracture surfaces of neat and PGF-reinforced thermosetting composites:

- Polyester without PGF: The fracture surface appears smooth and featureless, typical of brittle failure in unreinforced thermosetting polymers, indicating low energy dissipation.
- Epoxy without PGF: A relatively flat fracture surface with minor surface defects is observed, confirming the limited toughness and absence of reinforcing mechanisms.
- Polyester with chopped PGF (22.8 wt%): The micrograph shows randomly oriented and well-dispersed PGFs within the matrix. However, the presence of voids and visible fiber pull-out suggests partial interfacial bonding and limited stress transfer efficiency.
- Epoxy with chopped PGF (22.8 wt%): Improved fiber dispersion and stronger interfacial adhesion compared to the polyester matrix are evident. Fiber breakage and matrix tearing are observed, indicating a more effective stress transfer and enhanced toughness.
- Polyester with continuous PGF: The fracture surface reveals a well-aligned, unidirectional fiber arrangement, resulting from flow-induced orientation during contact molding. The strong fiber–matrix interface limits fiber pull-out, contributing to better mechanical performance.
- Epoxy with continuous PGF: A highly organized and compact fracture morphology is observed, with tightly packed, oriented fibers. Excellent interfacial bonding is demonstrated by minimal fiber debonding, indicating delayed failure mechanisms and superior mechanical resistance.

These morphological observations correlate well with the mechanical behavior of the composites. The neat resins (A, B) exhibit brittle fracture modes due to the absence of reinforcing elements. In contrast, the incorporation of chopped PGFs (C, D) improves fracture toughness, though the presence of local porosity—particularly in the polyester-based system—reduces the effectiveness of stress transfer. The continuous PGF composites (E, F) demonstrate the most favorable characteristics, with highly aligned fibers and strong interfacial adhesion, leading to superior mechanical properties such as higher tensile strength, stiffness, and energy absorption.

The differences in porosity between polyester and epoxy matrices can be attributed to their distinct curing mechanisms and fiber wetting behavior. The epoxy matrix, exhibiting better fiber wetting and lower shrinkage, results in a denser and more compact structure. Furthermore, the unidirectional alignment of the continuous PGFs, induced by the contact molding process, plays a crucial role in optimizing load transfer and crack propagation resistance during mechanical loading [35,36].

A summary of the composite configurations, fiber orientation, interfacial adhesion, and their expected impact on mechanical performance is provided in Table 7.

**Figure 9:**
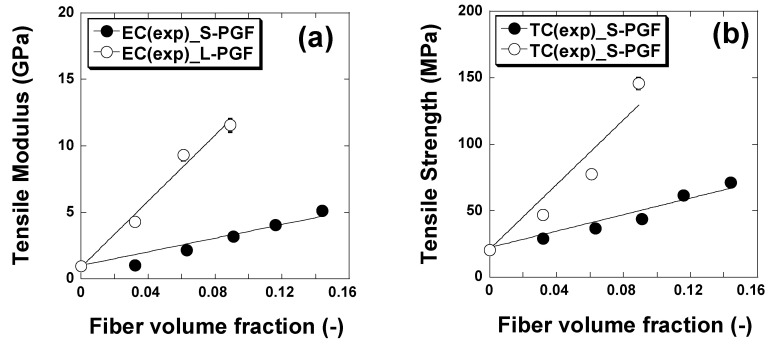
Comparison of experimental and theoretical tensile modulus (**a**) and tensile strength (**b**) of phosphate glass fiber-reinforced polyester composites. The solid lines correspond to the Hirsch model fit. S-PGF and L-PGF designate chopped and continuous phosphate.

**Figure 10:**
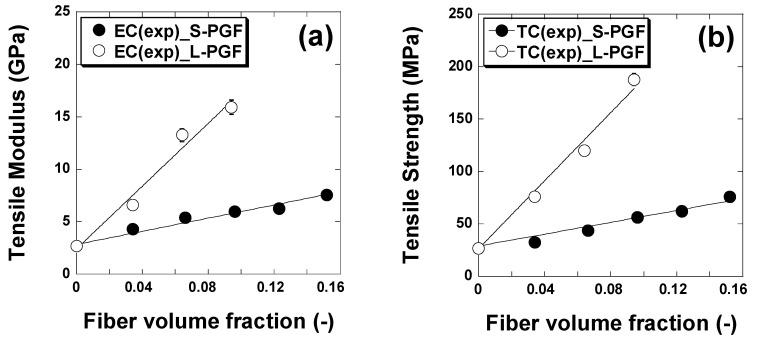
Comparison of experimental and theoretical tensile modulus (**a**) and tensile strength (**b**) of phosphate glass fiber-reinforced epoxy composites. The solid lines correspond to the Hirsch model fit. S-PGF and L-PGF designate chopped and continuous phosphate.

Theoretical values of tensile strengths and moduli of short and continuous phosphate glass fiber-reinforced epoxy and polyester matrices with different fiber volume loadings were calculated using the Hirsch model [35] and have been compared with the experimental values as reported in Figure 9 and Figure 10.

The Hirsch model is a combination of parallel (isostrain) and series (isostress) models of the rule of mixtures and is useful for estimating the mechanical properties of composites with randomly oriented fibers in a rigid matrix. The Hirsch model takes into account both the transverse tensile strength and the longitudinal tensile strength to calculate the overall tensile strength of the reinforced composites. The Hirsch model was selected since it is more suitable for predicting the mechanical properties of composites based on stiff fibrous reinforcements [35].

Equations (6) and (7) represent the Hirsch model of composites [35].

For the modulus of elasticity of composites E_c_,

(6) $$E_{c}=\beta \;\left(E_{m}\;V_{m}+{\;E}_{f}\;V_{f}\right)+\left(1-\beta \;\right)\;\frac{E_{f}\;E_{m}}{E_{m}\;V_{f}+E_{f}\;V_{m}}$$

For tensile strength of composites σ_c_,

(7) $$\sigma_{c}=\beta \;\left(\sigma_{m}\;V_{m}+\sigma_{f}\;V_{f}\right)+\left(1-\beta \;\right)\;\frac{\sigma_{f}\;\sigma_{m}}{\sigma_{m}\;V_{f}+\sigma_{f}\;V_{m}}$$

where E_f_ and E_m_ are tensile modulus, σ_f_ and σ_m_ are tensile strengths, and V_m_ and V_f_ are the volume fractions of the matrix and fibers, respectively.

The parameter β introduced in the Hirsch model characterizes stress transfer between the fiber and the matrix. It is controlled mainly by fiber orientation, fiber length (aspect ratio), and fiber/matrix interfacial strength. The coefficient β varies from 0 to 1. It is lower in the presence of composites with more randomly oriented fibers. β increases with increasing fiber length and the stress transfer between fiber and matrix. The latter could increase or decrease depending on the level of interaction between PGFs and epoxy/polyester matrices according to the chemical nature of the composite constituents. Note that when the parameter β is equal to unity, the Hirsch model coincides with the well-known rule of mixture (ROM) relationships assuming that both the matrix and fiber experience the same strain [36,37]. The ROM model works extremely well and is perfectly reasonable for aligned continuous-fiber composites with sufficient stress transfer from the matrix to the fiber. Indeed, Kretsis [38] indicated in their pioneering review paper that the longitudinal tensile modulus generally obeys the rule of mixtures (ROM) within experimental accuracy in continuous-fiber-reinforced composites due to strain compatibility through the thickness of the material.

In this work, the factor β was calculated by a curve-fitting routine over all the data to obtain best-fit values with the experimental results.

Figure 9 and Figure 10 show that tensile strength and elastic modulus increased regularly with the increase in the volume fraction of PGFs. A good agreement was observed between the theoretical and experimental values.

Table 7 depicts the calculated β coefficient from the fit of the experimental data using the Hirsch model. Regardless of the type of resin used (polyester or epoxy), it was found that in the case of chopped PGF-reinforced composites, the β parameter was close to 0.1, which is the coefficient value of stress transfer for randomly orientated fibers [37,38]. In the case of continuous phosphate glass fiber-reinforced composites, β coefficient values increased compared to S-PGF-based ones, indicating more parallel-oriented fibers. Nevertheless, in L-PGF/epoxy composites, the value of β was found to be much higher compared to L-PGF/polyester composites, approaching unity (i.e., equal strain in the two components), indicating effective stress transfer between phosphate glass fibers and the epoxy matrix, which was probably due to the strong interaction between the two components and the lack of porosity as demonstrated by SEM morphological analyses.

With R^2^ values all above 0.96, it is evident that the Hirsch model fits the experimental data quite well. Additionally, the data in Table 8 show that the errors between the experimental values and Hirsch model prediction are all less than 6%, indicating that the model has good predictive capability of tensile properties for PGF–polyester and PGF–epoxy composites.

It is useful to mention that the effect of voids within the composites has been neglected in the Hirsch and ROM models [39]. In the present work, the photomicrograph from failure surfaces presented earlier (Figure 8F) showed an intimate overall adhesion of L-PGF and epoxy resin but highlighted the presence of some microvoids between the fibers and matrix probably due to the fabrication process, and at the same time, the effectivity parameter β was close to unity (i.e., ideal situation), indicating little deviation from SEM observations. Iba et al. [40] found a similar β value in the case of a unidirectionally aligned continuous glass fiber-reinforced epoxy composite prepared by a hand lay-up process. The adjustable parameter β in the rule of mixtures was almost equal to unity. Dkier et al. [41] highlighted that in the case of woven glass fiber-reinforced polyphtalamide (PPA) prepared by a resin transfer molding (RTM) process, the critical parameter β was 0.986 despite the presence of some holes and bubbles in the micrographs. The latter were observed by analyzing the impregnation of inter- and intra-wicks from delaminated composite layers. Based on the same rule of mixture relationship, Rijsdijk et al. [42] indicated that fiber efficiency β is maximum (close to unity) in the case of continuous glass-fiber reinforced systems based on epoxy resin. On the other hand, β values were rather low in the presence of maleic-anhydride-modified polypropylene (m-PP) matrix composites. Maleic-anhydride-modified polypropylene was added to the PP homopolymer to improve the adhesion between the PP matrix and the continuous glass fiber, yielding values for β in the order of 0.85–0.95.

## 4. Conclusions

This study examines the physical, mechanical, and morphological properties of polyester- and epoxy-based thermoset composites reinforced with chopped and continuous glass fibers with a mass fraction ranging from 5.5% to 22.8%, which were elaborated on by the contact molding technique. The following conclusions can be drawn from this study:

- The addition of phosphate glass fibers to polyester–epoxy matrices results in a significant increase in composite density, particularly marked with chopped fibers. Epoxy, which is denser than polyester, strongly influences this increase, and continuous fibers contribute to increased density while reducing porosity, thus optimizing mechanical performance without altering structure. On the other hand, chopped fibers, due to their greater surface area, also modify density while influencing material rigidity.
- The studied composites exhibit improved thermal behavior, with initial thermal stability up to approximately 150–230 °C, depending on the fiber content, matrix type, and composite geometry. The incorporation of phosphate glass fibers and fillers, along with optimized structural design, contributed to a reduced rate of thermal degradation and a higher residual mass. These findings underscore the important role of reinforcement and formulation in enhancing thermal resistance and delaying decomposition.
- The addition of phosphate glass fibers to composites based on thermosetting resins, whether polyester or epoxy, significantly improves tensile and flexural strength, with a more marked increase in a continuous geometry than in a cut geometry. Phosphate glass fibers effectively reinforce both types of resin, although epoxy shows superior overall performance, particularly in terms of tensile and flexural strength, stiffness, and durability.
- Scanning electron microscopy (SEM) analysis highlighted the critical role of fiber–matrix compatibility and fiber configuration in determining the mechanical performance of the composites. Composites reinforced with continuous, well-aligned fibers exhibited excellent interfacial adhesion and optimized load transfer, resulting in enhanced mechanical strength. In contrast, chopped fibers, although easier to process, led to more limited improvements, highly dependent on adhesion quality—particularly low in polyester. These findings confirm that combining good fiber wetting with continuous reinforcement is essential to achieving high-performance composite materials.
- The experimental data from tensile strengths and Young’s modulus of s-PGF and L-PGF-reinforced polyester and epoxy composites were evaluated according to the theoretical composite Hirsch model. The results showed a good correlation between the experimental data and theoretical prediction with a high β interaction coefficient in the case of L-PGF-based epoxy composites compared to L-PGF polyester ones.

In summary, this study demonstrates the potential of phosphate glass fibers as promising reinforcement materials. To further advance this research, future work will focus on the surface treatment (sizing) of the fibers to improve interfacial bonding with polymer matrices. Additionally, efforts will be made to convert the fibers into woven and non-woven textile forms, which are more suitable for large-scale industrial applications, particularly in the automotive and aerospace sectors. These developments will not only enhance the versatility and processability of phosphate glass fibers but also increase their added value, enabling them to compete with conventional reinforcement fibers used in high-performance composite materials.

## Figures and Tables

**Table 1:** Properties of phosphate glass fiber [20].

Properties	Value
Density (g/cm^3^)	2.8
Tg (°C)	538
Tc (°C)	655
Young’s modulus (GPa)	140
Tensile strength (MPa)	2668

**Table 2:** Physical property evaluation of the composite materials developed with polyester.

Samples	Mass Fraction (%)	Composite Density (g/cm^3^)	Volume Fraction (%)	Thickness (mm)
Polyester/0% S-PGF	0	1.60	0	5.0
Polyester/5.5% S-PGF	5.5	1.64	3.2	5.0
Polyester/10.5% S-PGF	10.5	1.68	6.3	5.0
Polyester/14.9% S-PGF	14.9	1.71	9.1	5.0
Polyester/18.7% S-PGF	18.7	1.74	11.6	5.0
Polyester/22.8% S-PGF	22.8	1.77	14.4	5.0
Polyester/5.4% L-PGF	5.4	1.64	3.2	5.0
Polyester/10.2% L-PGF	10.2	1.67	6.1	5.0
Polyester/14.6% L-PGF	14.6	1.71	8.9	5.0

**Table 3:** Physical property evaluation of the composite materials developed with epoxy.

Samples	Mass Fraction (%)	Composite Density (g/cm^3^)	Volume Fraction (%)	Thickness (mm)
Epoxy/0% S-PGF	0	1.70	0	5.0
Epoxy/5.5% S-PGF	5.5	1.74	3.4	5.0
Epoxy/10.5% S-PGF	10.5	1.77	6.6	5.0
Epoxy/14.9% S-PGF	14.9	1.81	9.6	5.0
Epoxy/18.7% S-PGF	18.7	1.84	12.3	5.0
Epoxy/22.8% S-PGF	22.8	1.87	15.2	5.0
Epoxy/5.4% L-PGF	5.4	1.74	3.4	5.0
Epoxy/10.2% L-PGF	10.2	1.77	6.4	5.0
Epoxy/14.6% L-PGF	14.6	1.80	9.4	5.0

**Table 4:** Degradation ranges for composites based on phosphate chopped glass fibers and thermosetting epoxy and polyester resins.

Samples	Tmax	Residue (%) at Tmax
Epoxy/0% S-PGF	183	5
Epoxy/5.5% S-PGF	229	7
Epoxy/10.5% S-PGF	233	11
Epoxy/14.9% S-PGF	233	14
Epoxy/18.7% S-PGF	234	15
Epoxy/22.8% S-PGF	235	18
Polyester/0% S-PGF	187	0
Polyester/5.5% S-PGF	228	3
Polyester/10.5% S-PGF	235	7
Polyester/14.9% S-PGF	237	7
Polyester/18.7% S-PGF	239	10
Polyester/22.8% S-PGF	329	14.5

**Table 5:** Degradation ranges for composites based on continuous phosphate glass fibers and thermosetting epoxy and polyester resins.

Samples	Tmax	Residue (%) at Tmax
Epoxy/0% L-PGF	183	5
Epoxy/5.4% L-PGF	233	21
Epoxy/10.2% L-PGF	233	37
Epoxy/14.6% L-PGF	244	64
Polyester/0% L-PGF	187	0
Polyester/5.4% L-PGF	228	9
Polyester/10.2% L-PGF	228	34
Polyester/14.6% L-PGF	231	59

**Table 6:** Comparative study of the mechanical properties of glass fiber-reinforced polymers (GFRPs) and phosphate glass fiber-reinforced polymers (PGFRPs).

Resin	Choice of Geometry	V_f_	Tensile Strength (MPa)	Tensile Modulus (MPa)	Flexural Strength (MPa)	Flexural Modulus (MPa)	Ref.
Polyester	GFRP virgin	-	64.4	7200	-	-	[29]
	GFRP Chopped Strand	0.30	-	124	-	159	[26]
	GFRP woven carpet	-	220	7000	-	-	[30]
	Chopped strands + vertical rovings	-	103.5	-	-	-	[31]
	PGFRP chopped	15.2	71.3	5120	78.1	404	Present Study
	PGFRP continuous	9.4	145.7	11,550	167.9	547	Present Study
Epoxy	GFRP Randomly oriented	0.5	179.4	6700	297.8	-	[32]
	GFRP Unidirectional	0.55	784.9	-	-	-	[30]
	Woven FRP	0.60	311.0	18,610	-	-	[33]
	GFRP woven + (35% by weight short borosilicate)	-	355.0	43,700	-	-	[34]
	PGFRP chopped	15.2	75.9	7570	74.6	413	Present Study
	PGFRP continuous	9.4	187.9	15,930	218.0	786	Present Study

**Table 7:** Structure–mechanical property correlation.

Configuration	Fiber Orientation	Fiber/Matrix Adhesion	Expected Mechanical Properties
Neat matrices	None	–	High brittleness, low toughness
Chopped PGF	Random	Moderate (polyester), good (epoxy)	Moderate toughness, limited improvement in strength
Continuous PGF	Unidirectional	Strong (especially with epoxy)	High modulus, improved tensile strength, delayed crack propagation

**Table 8:** The β coefficient from the fit of experimental data using the Hirsch model.

PGF-Reinforced Composites	Fitting from Tensile Moduli Data			Fitting from Tensile Strength Data		
	β Coefficient	R^2^	Error	β Coefficient	R^2^	Error
Polyester/S-PGF	0.18	0.96	0.01	0.12	0.97	0.01
Polyester/L-PGF	0.89	0.99	0.04	0.46	0.96	0.05
Epoxy/S-PGF	0.22	0.98	0.01	0.11	0.98	0.01
Epoxy/L-PGF	0.99	0.99	0.06	0.61	0.99	0.03

## Data Availability

The raw data cannot be shared at this time as the data are also part of an ongoing study.
